# Policy Effects of Ecological Red Lines on Industrial Upgrading and Health Promotion: Evidence From China Based on DID Model

**DOI:** 10.3389/fpubh.2022.844593

**Published:** 2022-03-08

**Authors:** Penghao Ye

**Affiliations:** School of Economics, Hainan Open Economy Research Institute, Hainan University, Haikou, China

**Keywords:** Ecological Red Lines, industrial upgrading, health promotion, policy evaluation, difference-in-differences, event study

## Abstract

The implementation of the Ecological Red Lines (ERL) policy in China is under the background that natural resources have been immoderately exploited for serving rapid economic growth in the last 40 years, where the ecosystem's degradation happened and people's health could be affected. As the secondary industry is the contribution source of rapid growth as well as the threat source that threatens the natural environment and public health, the delimitation of ERL can act as a legal restriction that forces the industries to control the emissions and to upgrade the industrial composition. This paper conducts an ex-post policy evaluation on the improvement effects of industrial structure and residents' health and through ERL's pilot scheme in four provinces of China. By using the difference-in-differences (DID) method, the estimation results show that: (1) The industrial upgrading effect exists but to a small extent, as the ERL policy has generally elevated the tertiary industry's output by only 0.033% and hardly shown any promotion effects on the ratio of the tertiary industry to secondary industry; (2) The residents' health has been significantly improved by 1.029% after ERL policy on the whole, and enhanced over time mostly; (3) The health promotion effects are similar among three out of the four pilot provinces, whereas the industrial upgrading effects performed large heterogeneities among the four. These empirical results may provide references for the wider extension of ERL policy with more practical execution solutions in developing economies.

## Introduction

A typical developing region is composed of cities, villages and towns normally distributed with secondary industries. The secondary industries normally produce the manufactured or intermediate goods for domestic and global supply chains. By transaction and usage of the goods, the residents' welfare and economic growth can be promoted. However, while commercial manufacturing can provide intuitive sizable economic statistics, the consumed natural resources, including land, clean water, forest, mines, non-renewable energy sources, and biodiversity, have been neglected or regarded as slight “negative externality” for a long period (1, 2), even though they served as indispensable elements for secondary industry (3). As a result, people's health could be negatively affected by the diminishing resources and contaminated environments. Because of the manufacturing-dominated industrial structure and the production-oriented growing demand, this situation appears to be more widespread in developing countries compared with developed economies which finished the industrial transformation in the main (4–6). As a consequence, the residents' health degree could come down and the industrial structure remains pollution-intensive and energy-intensive, causing a greater loss for both the individuals and the whole society. According to the Food and Agriculture Organization of the United Nations (FAO), the global resources of soil, land and water is “reaching the limit,” as the land area per capita declined by 20% from 2000 to 2017, the groundwater consumption rose from 688 km^3^/year in 2010 to 820 km^3^/year in 2018 and let to a 250 km^3^/year of water loss for the soil aquifer, the globe will suffer from a higher risk of environmental disasters and prevalence of disease if the current consumption trends have not ceased (7).

This circumstance began to change since the environmental and illness events continually occurred in multiple developing regions, as the adverse effects of heavy-polluting industries became more noticeable and received broader concerns in the societies. To contain the frequent ecological hazards, some developing countries have begun to take environmental protection measures. In order to protect the biological diversity, South Africa has built 422 ecological preservation zones with a total area of 67,000 square kilometers, where much stricter environmental laws and higher farm chemical standards are executed (8); In Brazil, a construction project of the potential world's third-largest hydropower station with a capacity of 11.3 gigawatts on a tributary of the Amazon River in Belo Monte came to a halt in 2012, for the reason that the submerged rainforest upstream of the dam may infringe the local biodiversity, especially for the species of fish and bird (9, 10). These actions indicated that the emerging economies are attaching more importance to ecological protection, despite economic growth still being the primary goal.

China is the second-largest economic entity as well as the biggest developing country around the world currently, experiencing a spectacular increase of production capacity in the last two decades, but also accompanied by an enormous depletion of natural resources and environment due to the rapid expansion of secondary industries. During the booming period of foreign trade (2001–2009) and infrastructure construction (2005–2014), environmental pollution and climate change events became more frequent and extensive than before. For the storm-flood event, few Chinese cities had encountered it before 2001, but the waterflood came out many more times since then, where 60% of the cities in China have witnessed the happening of storm-flood in 2015 (11). For the air quality issues, the annual haze pollution event in northern China increased from 30–50 days in 2000 to 80–90 days in 2006, and maintained at a level of 70–90 days until 2014 (12); some parts of southern China have also been intruded by winter haze caused by El Niño events since 2,000 (13). As for the water quality, every 1% transfer of the state-owned land transfer could bring 0.08–0.113% agriculture wastewater discharge at the prefecture-level (14), and the water pollution in rural areas was even much worse (15). All these environmental pollution events can threaten the residents' health to varying degrees by environmentally related diseases or declining food quality, then the wellbeing of most families would be deprived as a consequence.

Apart from the environmental issues, the resource depletion in manufacturing-based China is also tremendous. Taking energy consumption as an example, China consumed an average of around 4,000 megaton coal in 2011–2015, accounting for half of the world's consumption, and was five times as much as the consumption in the US. The electricity consumption per GDP in China was 0.508 kilowatt/USD in 2015, which was 2.28 times as Japan's and 4.65 times as UK's in the same year (source: World Bank); In terms of thermal unit, China's energy intensity was about 7.60–9.19 kBtu/USD in 2011–2015, which was 50.2–67.5% higher than the world average level (source: International Energy Agency). One reason accounting for this high consumption is the “world factory” status of China, as China has become the world's biggest exporter for trade in goods since 2009, accompanied by high production demands for both the domestic and the foreign market. Another reason is the coal-based energy structure and energy-intensive industries: China can produce 7,128.2–8,306.3 billion megajoule of raw coal annually, but the annual yield of the crude oil and natural gas are 834.7–896.8 billion and 373.6–533.8 megajoule; the secondary industry shared more than 40% of gross output before 2015 (source: National Bureau of Statistics of China), which was doubled compared to the developed countries averagely.

These intermittent environmental and climate events, along with continuous high resource consumption, aroused great concern from the Chinese government and residents, a plentiful of anti-pollution measures and environmental regulations have been carried out hard upon. Being different from other countermeasures that are directed at one or several specific pollutants or greenhouse gas, the Ecological Red Lines (ERL) policy is an all-around ecological and environmental action that limits the misuse of resources and immoderate emissions. The Ministry of Ecology and Environment (MEE) of China introduced this policy in 2015, and it can also be called the Ecological Conservation Red Lines.

**Figure 1:**
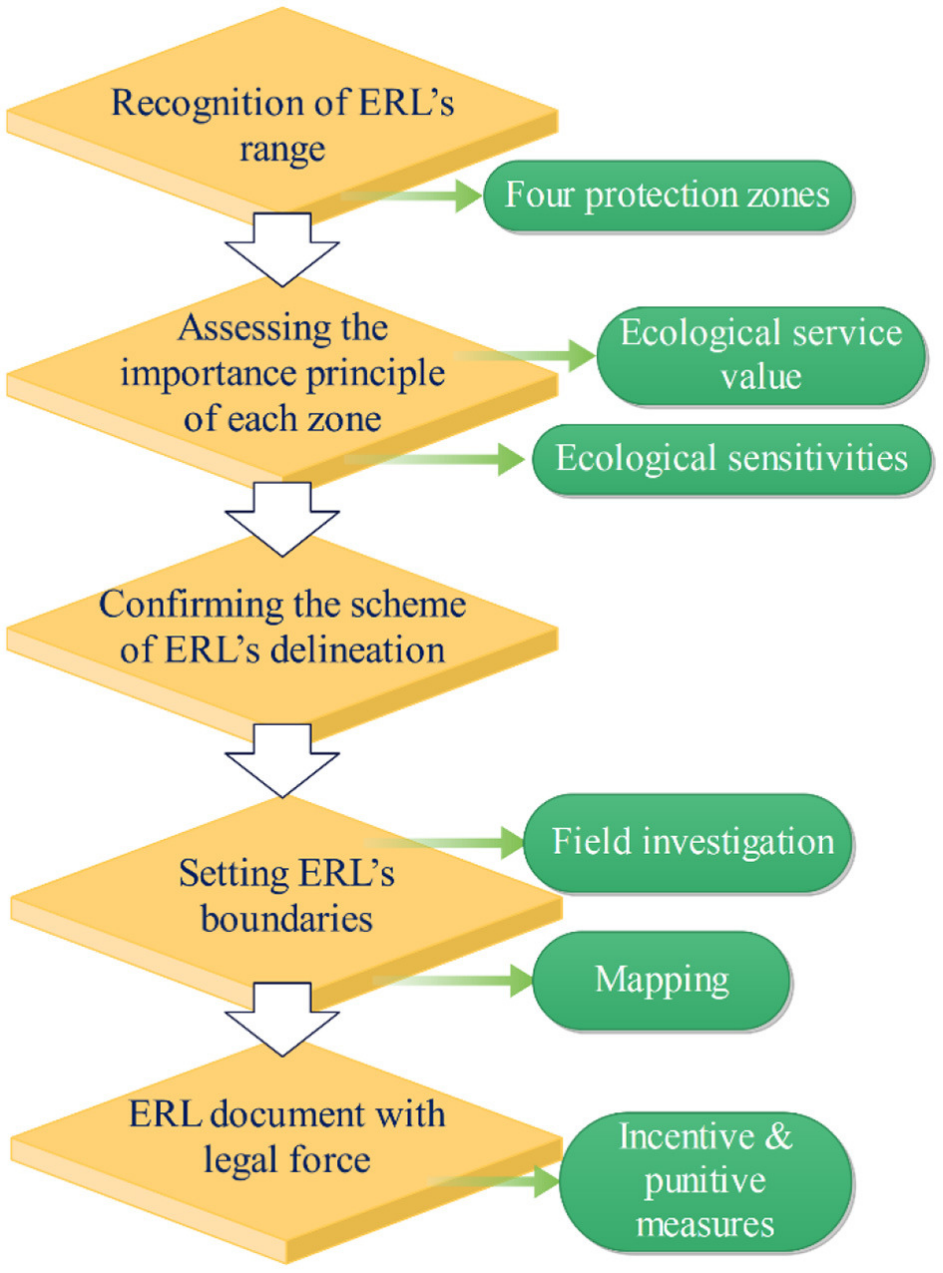
Formulation process of ERL policy.

According to Figure 1, the delimiting of the ERL can is carried through with five main steps. The initial step is the recognition of the ERL's range, in which four protection zones are divided (see **Figure 3**). The subsequent step is assessing the importance principle of each zone in terms of service value and sensitivities of the ecosystems. Based on the assessment results, the scheme of ERL's delineation and its boundaries will then be confirmed and set. Finally, the formal ERL document with legal force will come into force, preventing the ecological protection areas from improper exploitations.

**Figure 2:**
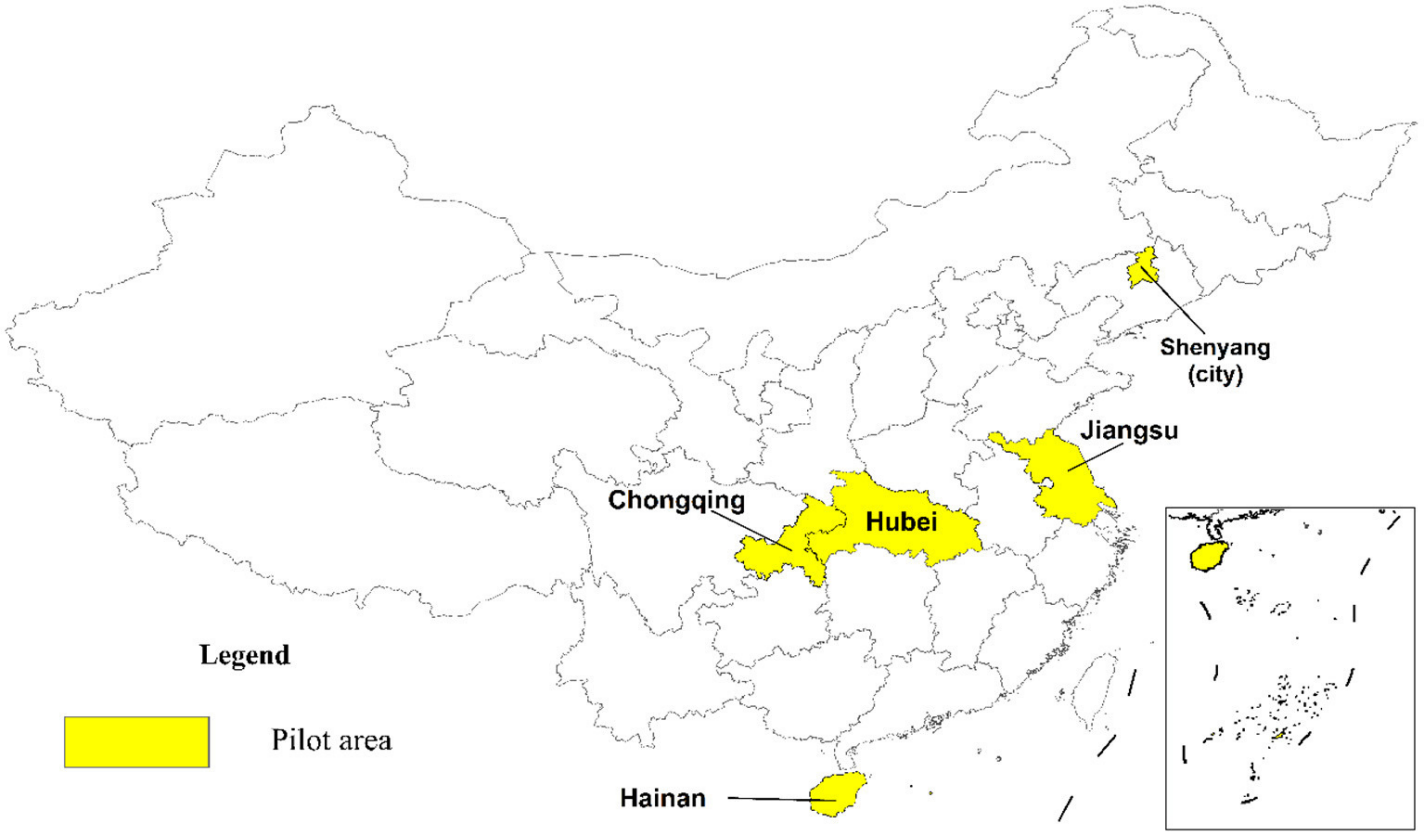
The pilot regions for ERL implementation in 2015.

The concept of ERL was officially proposed in 2012 when the MEE launched the formulation of ERL. Subsequently, from 2012 to 2014, more refined concepts and more detailed management practices of ERL have deepened, as some city-level pilot programs were attempted and offered valuable experiences useful for the follow-up policies enactment making of ERL for MEE (16). Based on the above efforts and investigations, a policy mix was released in 2015 (17), that is, the official document *Technical Guide for Delimiting Ecological Protection Red Lines* (18) issued by MEE, then four provincial-level regions and one city-level region were formally selected as the pilot areas for the implementation of ERL (shown in Figure 2), where the ecological preservation areas were defined with strict supervisory policies.

As Figure 2 indicates, four provinces, Jiangsu, Hubei, Hainan and Chongqing, with Shenyang city, are the pilot regions for the ERL's trial implementation. Each administrative unit has a representative feature in China: Jiangsu is a typical advanced eastern coastal province, Hubei is a medium-developed inland province in central China, Hainan is a tropical island at an economic take-off stage and fit for wide-range reforms, Chongqing is a provincial-level mountainous municipality shouldered with poverty alleviation tasks, Shenyang is a northeastern provincial capital city with central heating in winter. Within the ERL zones of these pilot areas, environmental entry, ecological performance assessment, ecological compensation, emission control and other rigid protection measures were strictly put into practice.

**Figure 3:**
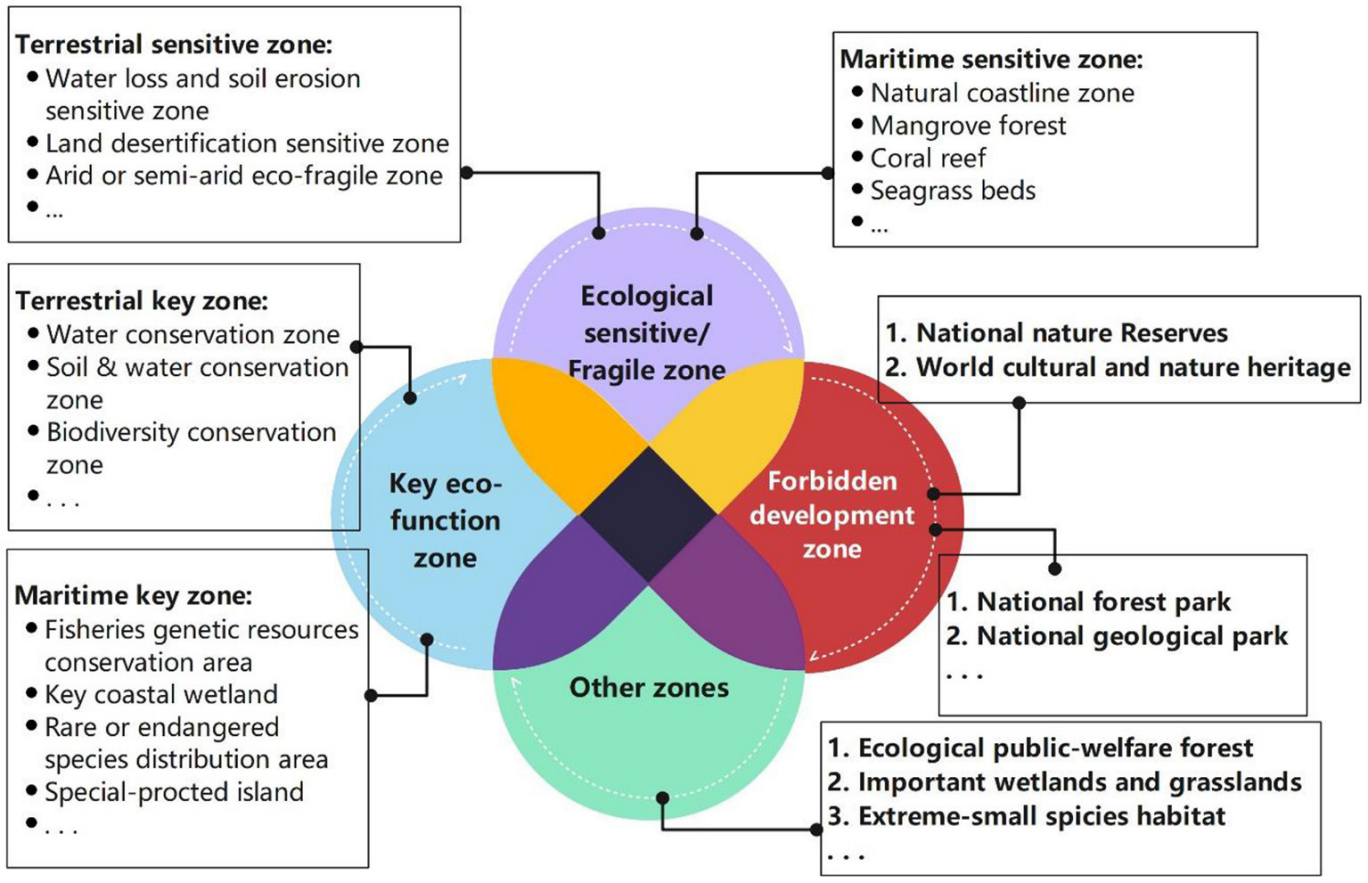
Diagram of the ERL's delineation.

According to the technical guide of the ERL pilot policy (18), four ecology protection zones constitute the ERL zones in Figure 3: key eco-function zone, ecological sensitive/fragile zone, forbidden development zone, and other zones (e.g., Ecological public-welfare forests, key wetlands). Each zone was treated differently in management details, but they may have intersections of coverage, as they have common features including the limitation of land development, resource exploitation, and emission dumping. In these zones, four control requirements are strictly executed: the land usage must not be transformed, the ecological function must not be lowered, the area of the zone must not be reduced, and the protection responsibilities of the forest, grassland, wetland and wilderness must not be changed. Also, ecological restoration is another important target in the red line areas, as they may have been damaged by industry and agriculture expansion in the past two decades before 2015.

Based on these actions, it is noteworthy to evaluate what has ERL achieved in multiple aspects. Focused on four pilot provinces shown in Figure 2^1^ and by using the policy evaluation econometric models, this paper chooses the industrial upgrading as the direction index for assessing the direct effect of the ERL policy, and the health promotion as the indicator for investigating the indirect effect, aiming at finding out the effect significance, the effect degree, and the possible regional heterogeneities of the ERL. Moreover, an event study applied to discovering the year-by-year effect will be carried out if the result shows high significance and extensiveness among the four pilot provinces both as a whole or individually.

## Literature Review and Hypothesis Proposing

### Existing Studies on ERL

Although the notion of ecological protection came a long time ago and relevant policies and actions had been taken in many countries or regions, the concept of the Ecological Red Line was first proposed in China in 2005 but remained at the discussion stage for over a decade (19). During the period of discussion, the academic expectation emerged before the ERL came to trial implementation. Si et al. pointed out that systematic delimitation, scientificity and maneuverability were the main principles for formulating the marine regional ERL (20). Based on the potentialities of ERL's functions, Lu et al. made a comparison study on the traditional Marine functional zoning (MFZ) and more recent Marine ERL, discovering that the Marine ERL can strengthen the MFZ by more specified regulations on administrations (21). As the basic execution unit of ERL is a province, Wang et al. assigned different indices with different weightiness in China's Liaoning Province, emphasizing the fundamental role of ecological inventory for the dynamical protection of the ERL.

Because the ERL is a dynamic policy that needs continuous improvement, several scholars have put forward their understandings and suggestions on how to effectively delimit its scope and strengthen its protection measures. Xu et al. conducted a case study on the Yangtze River Economic Belt which covers 11 provinces or municipalities, identifying that the key indicators, the evaluation methods, and the synergetic protection with other protection zones are the three knowledge gaps of the understanding of ERL; therefore, standardization, independent professional agencies, third-party evaluation systems, and cross-provincial ecological compensation strategies are four appropriate countermeasures for the implementing of ERL (16). He et al. unscrambled the ERL pilot policy in 2015 by classifying four types of ERL zones into more sub-zones with different functions and distinguishing the implementation points of land ERLs and maritime ERLs, then proposed that the financial support is a long-term guarantee of ERL's maintenance (22). The literature above has interpreted the ERL policy or provided the evaluation criteria of the ERL, but hardly examined the implementation effects of the ERL pilot policy. Hence, this paper chooses the industrial upgrading and residents' health as the main indicators, attempting to find out whether the ERL policy can markedly reduce the sickness rate in the pilot provinces from 2015 to 2019.

### Policy Evaluation Method

Policy evaluation, or policy assessment, is a prevalent research method in empirical econometric studies, these methods include the instrumental variable (IV) method, regression discontinuity design (RDD), synthetic control method (SCM), and difference-in-difference (DID) method. The IV method can effectively solve the endogenous problem in causal analysis (23), but the appropriate selection of IVs and the excluding of the study objects' heterogeneities are not easy to overcome (24). The RDD became widespread since Hahn et al. provided a comprehensive theoretical framework (25), but its high requirement of geographical homogeneities could restrict the evaluation of multiple observation subjects scattered in different regions (26). The SCM can give an intuitive graphical result displaying the trend variation before and after the policy implementation (27), but lacks quantitative outcomes and has difficulties in constructing complicated models (28). Among the methods for policy evaluation, the DID approach can provide the quantified results while overcoming the disturbance from the unobserved confounders (29). Ashenfelter came up with the earliest concept of DID (30), and Heckman et al. firstly put the DID method into policy assessment (31, 32). The DID model has served as an extensive tool in various kinds of research fields since then and was developed into multiple derivative models by scholars. In the field of environment and climate change, Greenstone and Hanna adopted DID estimator to find the distinctive results of the air pollution control acts and the water pollution control act in India (33). Lin et al. (34) used this approach to calculate out that greenhouse gas in China can be reduced by 1.75% annually due to the opening of high-speed railways. Nunn and Qian (35) extended their functions to estimate more than a single impact through multiple-period difference-in-differences (35).

DID method has also been widely used in the research field of public health for inspecting the achievements of a health policy or evaluating the impact of a pandemic. Dimick and Ryan summarized the use of DID in the assessments of health policies, believing that it is a better approach to solve the unpredictable background changes than the observational studies (36). Agüero and Beleche used DID to explore the health shocks and the long-lasting influences brought by the 2009 H1N1 influenza pandemic in Mexico, finding that although the H1N1 had caused health losses, the people's health was improved in the long run, owing to the awareness of personal hygiene has been promoted in a wider range (37). Li et al. adopted DID method to estimate the effect of lock-down during the prevention and treatment period of COVID-19, indicating that the citizen's short-term health can be improved due to the temporary improvement of the air quality (38).

### Hypothesis

**Figure 4:**
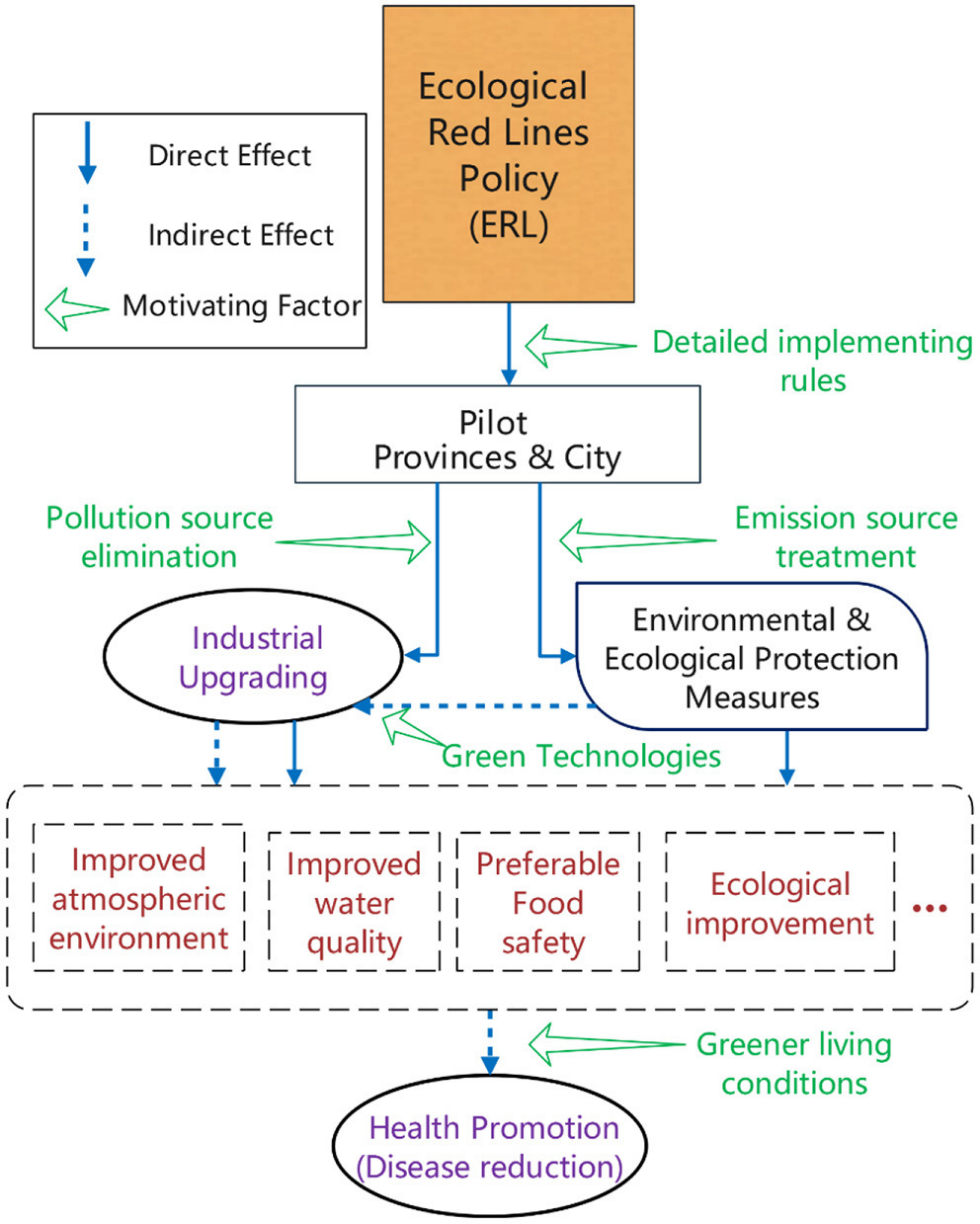
A Route map of ERL's conducting effects.

As mentioned before, the observation of two important indicators, industrial upgrading and health promotion, may reflect the effectiveness of the ERL pilot policy. To verify these effects, this paper presents a mechanism that the ERL may act on, as Figure 4 displays:

Figure 4 depicted the ERL's possible influence paths on the two indictors (in ovals), in which the industrial upgrading is directly affected. Considering this direct path, the industrial upgrading which turns the secondary industries into tertiary industries can be seen as a direct reaction to the environmental policy for the pilot provinces. Hence, a hypothesis (H_1_) that reflects the direct feedback of the ERL can be proposed as:

**H**_**1**_: *The ERL policy can significantly urge the pilot provinces to carry through industrial transformation by promoting the service industry's scale and its ratio to secondary industry*.

Compared to those observable or measurable indices such as forestry area, PM2.5, chemical oxygen demand (COD), or the three industrial wastes (wastewater, waste gases, and residues), the positive indirect effects of ERL policy are worth evaluating as well. This is why empirical studies based on panel data and econometric models are of use for discovering the indirect effects.

Figure 4 also describes a potential route that changes the health conditions. The ERL policy changed the industrial distribution where fewer eco-spaces were occupied; the environmental and ecological protection measures, which is another direct action that paralleled to industrial upgrading, would be taken to reduce the emission of hazardous substances. During these two direct reactions of the ERL, atmospheric environment and water quality would be improved as a result, food safety could be ensured, and more green technologies may contribute to the cleaner industrial production since then. Finally, the residents' health conditions could be promoted as a consequence of a cleaner ecological environment physically and psychologically, which can be seen as an indirect effect from the ERL as a whole. Hence, hypothesis 2 (H_2_) is put forward as follows:

**H**_**2**_: *The ERL policy can significantly improve the residents' health in the pilot provinces by reducing the number of disease-infected people*.

## Method

### Model Principle

**Figure 5:**
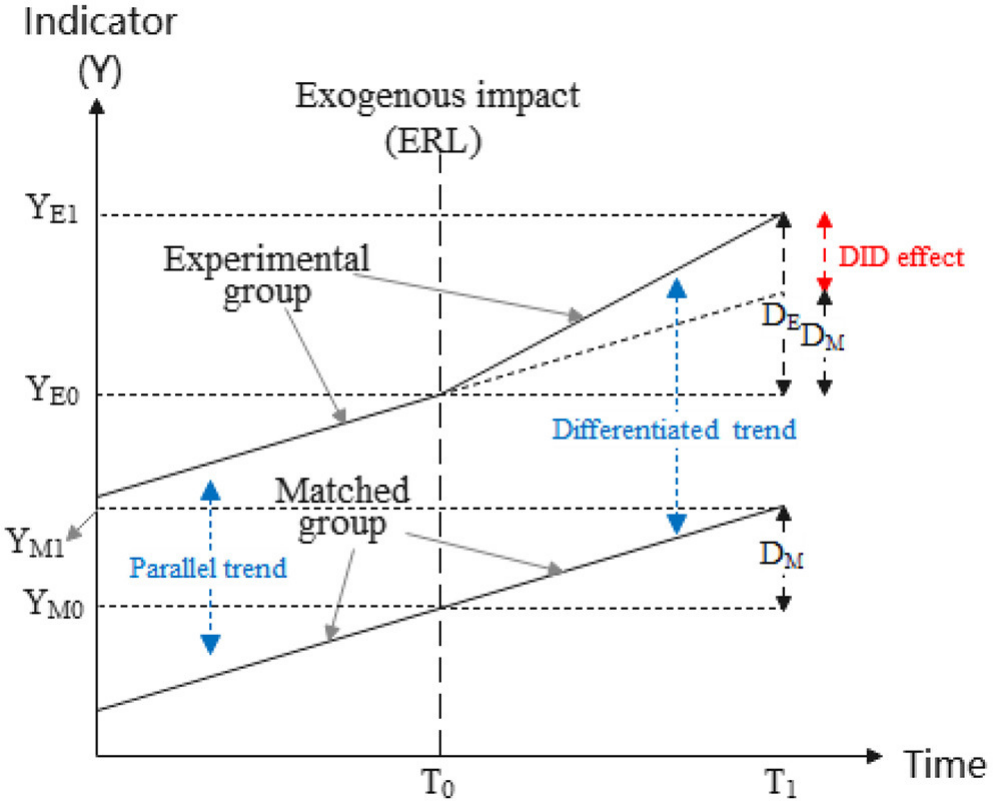
Evaluation principle of DID method.

The DID method is suitable for evaluating the trend differentiation of an object affected by an exogenous impact (e.g., A pilot policy), where the object can be a single entity or a group of individuals. Similar to a scientific experiment, the affected object can be identified as the experimental group or the treated group, whereas the other unaffected individuals can be regarded as the matched group or the controlled group, as shown in Figure 5.

The most critical concern is the trend differentiation of the indicators between the experimental group and the matched group after the exogenous impact. Figure 5 labeled the indicator's variation of the experimental group as D_E_, and that of the matched group as D_M_, thus D_E_ and D_M_ can be calculated by a first-time difference as:


(1) $$\begin{array}{l} D_{E}=Y_{E1}-Y_{E0}=E\left(Y_{E1}|t=T_{1}\right)-E\left(Y_{E0}|t=T_{0}\right) \end{array}$$

(2) $$\begin{array}{l} D_{M}=Y_{M1}-Y_{M0}=E\left(Y_{M1}|t=T_{1}\right)-E\left(Y_{M0}|t=T_{0}\right) \end{array}$$

Where the T_0_ and T_1_ in (1) and (2) are the start time and the cut-off time for observation of the exogenous impact. Therefore, the DID effect that indicates the differentiated trend at the observation period (T_0_-T_1_) can be figured out by a second-time difference as:


(3) $$\begin{array}{l} DID\;effect=D_{E}-D_{M} \end{array}$$

Besides, the prerequisite of a feasible DID estimation is that the experimental group and the matched group are sharing a common trend before the exogenous impact (39), that is, they have a parallel trend before T_0_. Hence, the parallel trend test will be carried through after the empirical tests.

### Empirical Model Specification

In this study, the implementation of the ERL pilot policy is treated as the exogenous impact, the experimental group consisted of the four pilot provincial units in China, which are Jiangsu, Hubei, Hainan, and Chongqing, whereas the matched group is composed of other provinces without ERL policy (even in city-level) before the cut-off time and have the regional homogeneities with the former. The implementation time T_0_ is 2015 when the ERL pilot policy was put into effect, and the cut-off time T_1_ of ERL is 2019 due to the dataset availability.

To estimate the industrial upgrading effect of the ERL pilot policy (verifying H_1_), the DID models are set as:


(4) $$\begin{array}{l} ln\left({Tertiary}_{it}\right)=\alpha +\beta \cdot \left({ELR}_{i}\times {Post}_{t}\right)+\overset{n}{\underset{j=1}{\sum }}\left(\gamma_{j}\cdot {Control}_{it}\right)\\ \;+\delta_{i}+\mu_{t}+\varepsilon_{it} \end{array}$$

(5) $$\begin{array}{l} {Update}_{it}=\alpha +\beta \cdot \left({ELR}_{i}\times {Post}_{t}\right)+\overset{n}{\underset{j=1}{\sum }}\left(\gamma_{j}\cdot {Control}_{it}\right)\\ \;+\delta_{i}+\mu_{t}+\varepsilon_{it} \end{array}$$

Where in model (4), the explained variable (dependent variable) *ln*(*Tertiary*_*it*_) indicates the logarithmic value of tertiary industry's output (production value of service sector) at province *i* in year *t*. The explained variable *Update*_*it*_ in model (5) refers to the ratio of the tertiary industry's output to the secondary industry's output at province *i* in year *t*. Considering the industrial upgrading indexes are multitudinous but the tertiary industry is the core factor among all of them (40), its direct output value and its ratio value to the traditional secondary industry are both adopted to reflect the comprehensiveness of the empirical study. β is the estimated coefficient of this study's concern, as its significance will prove the effectiveness of the ERL policy, and its value will reflect its effect degree. The policy dummy variable *ELR*_*i*_ is a binary variable denote to whether the ERL policy had been executed in province *i*, where it takes the value 1 if the province *i* had executed ERL and takes zero otherwise. The time dummy variable *Post*_*t*_ is also bivariate with 1 or 0, where it takes 1 when the pilot year of ERL begins (that is, *t* ≥ 2015) and equals zero before that (*t* < 2015). The *Control*_*jt*_ in the sum term are controlled variables that may have a relationship with the dependent variable but are unaffected by the policy (41), consisting of the real estate investment [in logarithmic form as ln(*Estate*_*jt*_)] and the energy consumption [in logarithmic form as ln(*Energy*_*jt*_)] in model (4) and (5), as they serve as the normal contributions to industrial development in immense amounts (42).

The indirect effect is also worth estimating, that is, the health promotion of the residents in the pilot provinces. To estimate this indirect effect of ERL pilot policy, the DID model is set accordingly as:


(6) $$\begin{array}{l} ln\left({Disease}_{it}\right)=\alpha +\beta \cdot \left({ELR}_{i}\times {Post}_{t}\right)+\overset{n}{\underset{j=1}{\sum }}\left(\gamma_{j}\cdot {Control}_{it}\right)\\ \;+\delta_{i}+\mu_{t}+\varepsilon_{it} \end{array}$$

Where the dependent variable *ln*(*Disease*_*it*_) in model (6) represents the logarithmic value of disease-infected people with their premarital examination at province *i* in year *t*, which is an indicator that delegates the residents' health. The controlled variables here are the number of health personnel [in logarithmic form as *ln*(*H*.*Personnel*_*it*_)], and the total assets of medical and health organization [in logarithmic form as *ln*(*M*.*H*.*Asset*_*jt*_)], considering that these two components can affect the dependent variable but has no relationship with the policy implemented (41, 43). The other coefficients and terms have the same meaning as those in model (4) or (5).

In consideration of the health data availability, the choice of the explained variable in model (6) can keep off the chronic disease primarily infected by the elders, which may bring the lagged elements or even deviations of the estimation results. As the newly-married people in China are mainly under 35, their health conditions can reflect the effects of the ecological environment much more explicitly. Moreover, the controlled group in model (4), (5) and (6) are all provincial units in China, but Liaoning Province is excluded as its capital city Shenyang is also the ERL pilot region. Finally, considering the inherent regional difference in four pilot provinces, each model will be estimated 5 times, including one merging effect and four individual effects on each of them.

### Data Source and Descriptive Statistics of Variables

In this empirical study, the data in the model (4), (5) and (6) are all collected from the EPS China database website (44), where the statistics are all legally collected from the National Bureau of Statistics of China, National Health Commission of P.R.China, and National Energy Administration of China. Table 1 presents the descriptive statistics of all the datasets to be analyzed.

**Table 1:** Descriptive statistics of variables.

**Variable**	**Obs**	**Mean**	**Std**	**Min**	**Median**	**Max**	**Unit**
*DID = ERL × post*	150	0.133	0.341	0.000	0.000	1.000	–
*Tertiary*	150	930.868	866.150	56.740	725.190	5,085.210	Billion CNY
*Update*	150	1.157	0.393	0.527	1.098	2.923	–
*Estate*	150	3.88 × 10^6^	1.08 × 10^7^	108.19	3,057.38	5.67 × 10^7^	100 million CNY
*Energy*	150	1.35 × 10^4^	7,540.005	1,358.507	1.26 × 10^4^	3.25 × 10^4^	Million ton of standard coal
*Disease*	150	21,463.520	26,644.912	9.000	7,050.500	1.09 × 10^5^	Person-time
*H.Personnel*	150	342.351	212.536	35.224	291.936	887.78	Thousand people
*M.H.Asset*	150	10,740.763	7,686.491	727.686	9,030.770	35,246.375	Billion CNY

All statistics above are panel data that are suitable for the econometrics regression analysis for model (4), (5) and (6), where 26.7% of them belong to the experimental group and the rest 73.3% are from the matched group.

## Results and Discussion

### Estimation of Industrial Upgrading

Setting tertiary industry's output as the explained variable in model (4) and the ratio of tertiary industry's output to secondary industry's output in model (5), the DID estimation results of the ERL policy on industrial upgrading are shown in Table 2, where the overall effect among the four pilot provinces, and the individual effect on every single province, are all included^2^.

**Table 2:** Policy estimation results of industrial upgrading.

**Explained variable**	**Overall *ln (Tertiary)* (1-a)**	**Overall *Update* (1-b)**	**Jiangsu *ln (Tertiary)* (2-a)**	**Jiangsu *Update* (2-b)**	**Hubei *ln(Tertiary)* (3-a)**
*DID* = *ERL*_i_ × *post_*t*_* ($\hat{\beta }=$)	0.033* (1.95)	0.027 (0.67)	0.003 (0.10)	−0.048 (−0.93)	0.066** (2.06)
*ln(Estate)* (${\hat{\gamma }}_{1}=$)	0.104** (6.03)	−0.014 (−0.33)	0.102*** (5.43)	−0.015 (−0.631)	0.108*** (2.90)
*ln(Energy)* (${\hat{\gamma }}_{2}=$)	−0.174 (−3.31)	−0.281** (−2.17)	−0.164** (−2.62)	−0.734*** (−6.92)	−0.189*** (−3.00)
Constant ($\hat{\alpha }=$)	9.217*** (15.39)	4.293*** (2.92)	9.210*** (12.78)	9.515*** (7.80)	9.373*** (13.01)
Time fixed effects	Yes	Yes	Yes	Yes	Yes
Region fixed effects	Yes	Yes	Yes	Yes	Yes
Observations	150	150	120	120	120
R^2^	0.987	0.762	0.985	0.855	0.986
**Explained variable**	**Hubei** ***Update*** **(3-b)**	**Hainan** ***ln (Tertiary)*** **(4-a)**	**Hainan** ***Update*** **(4-b)**	**Chongqing** ***ln*** ***(Tertiary)*** **(5-a)**	**Chongqing** ***Update*** **(5-b)**
*DID* = *ERL*_i_ × *post_*t*_* ($\hat{\beta }=$)	−0.112** (−2.08)	0.032 (0.99)	0.424*** (6.47)	0.026 (0.81)	−0.215*** (−3.85)
*Ln* *(Estate)* (${\hat{\gamma }}_{1}=$)	−0.015 (−0.48)	0.104*** (5.51)	−0.020 (−0.52)	0.105*** (5.54)	−0.017** (−0.53)
*Ln* * (Energy)* (${\hat{\gamma }}_{2}=$)	−0.724*** (−6.87)	−0.162* (−2.54)	−0.664*** (−5.18)	−0.171*** (−2.75)	−0.693*** (−6.39)
Constant ($\hat{\alpha }=$)	9.365*** (7.76)	8.834*** (12.37)	8.683*** (6.02)	9.141*** (12.89)	9.008*** (7.29)
Time fixed effects	Yes	Yes	Yes	Yes	Yes
Region fixed effects	Yes	Yes	Yes	Yes	Yes
Observations	120	120	120	120	120
*R* ^2^	0.856	0.985	0.837	0.986	0.843

***, **, and * means the statistical significance is at the 1, 5, and 10% accuracy level. The values in the brackets are the t statistics for each regression.

In the mass, the results from Table 2 indicate that the industrial upgrading effect brought by the ERL is effective but insufficient, as the output value of tertiary (*Tertiary*) industry has increased 0.033% by the reason of the EPR policy [in column (1-a)]. Moreover, the insignificant estimated coefficient in column (1-b) indicates that the ratio of tertiary industry's output to secondary industry's output (*Update*) has not been proved in connection with the ERL policy.

Moreover, the provincial-level results of industrial upgrading reveal the regional heterogeneity to a great extent, because the significances and values of their DID coefficients $\hat{\beta }$ have been varied by and large. The ERL policy has no significant effect in Jiangsu Province completely [see the $\hat{\beta }$ in column (2-a) and (2-b)], has a positive effect on *Tertiary* but negative on *Update* in Hubei [in (3-a), (3-b)]; for Hainan and Chongqing, both their *Update* terms are significantly related to the ERL but in the opposite direction [see (4-b) and (5-b)] and other indicators are insignificant [(4-a) and (5-a)], where Hainan's industrial upgrading ratio *Update* is positive and that of Chongqing is negative [(4-b) and (5-b)]. These differentiated results may reflect the different actions in each pilot province due to their own situations, which will be discussed further in 4.3.

In summary, the industrial upgrading effect of the ERL exists but with limitations, therefore hypothesis H_1_ is only partially true.

### Estimation of Health Promotion

In order to estimate the health promotion effect from the ERL policy, the number of disease-infected people with their premarital examination in model (6) is treated as the explained variable. The DID estimation results are listed in Table 3 as follows.

**Table 3:** Policy estimation results of health promotion.

**Explained variable**	**Overall (1-1)**	**Overall (1-2)**	**Jiangsu (2-1)**	**Jiangsu (2-2)**	**Hubei (3-1)**
*DID* = *ERL*_i_ × *post_*t*_* ($\hat{\beta }=$)	−0.793* (−2.07)	−1.029** (−2.72)	−1.063* (−4.40)	−1.319** (−2.37)	−0.061 (−0.25)
*Ln* * (H.Personnel)* (${\hat{\gamma }}_{1}=$)	–	4.391*** (3.47)	–	4.474** (2.89)	–
*Ln* * (M.H.Asset)* (${\hat{\gamma }}_{2}=$)	–	−0.191 (−0.11)	–	0.065 (0.03)	–
Constant ($\hat{\alpha }=$)	7.830*** (51.68)	−43.970** (16.952)	7.636*** (27.09)	−48.367** (−2.53)	7.449*** (26.90)
Time fixed effects	Yes	Yes	Yes	Yes	Yes
Region fixed effects	Yes	Yes	Yes	Yes	Yes
Observations	150	150	120	120	120
*R* ^2^	0.414	0.469	0.456	0.517	0.482
**Explained variable**	**Hubei** **(3-2)**	**Hainan** **(4-1)**	**Hainan** **(4-2)**	**Chongqing** **(5-1)**	**Chongqing** **(5-2)**
*DID* = *ERR*_i_ × *post_*t*_* ($\hat{\beta }=$)	−0.189 (−0.89)	−1.544*** (−6.39)	−1.671*** (−0.88)	−0.503* (−2.08)	−0.946 (−4.34)
*ln(H.Personnel)* (${\hat{\gamma }}_{1}=$)	5.094** (2.90)	–	4.968** (3.08)	–	4.830** (2.92)
*ln(M.H.Asset)* (${\hat{\gamma }}_{2}=$)	−0.360 (−0.17)	–	−0.369 (−0.17)	–	−0.338 (−0.15)
Constant ($\hat{\alpha }=$)	−51.540** (−2.54)	7.474*** (26.66)	0.275*** (17.09)	7.385*** (26.70)	−48.285** (−2.54)
Time fixed effects	Yes	Yes	Yes	Yes	Yes
Region fixed effects	Yes	Yes	Yes	Yes	Yes
Observations	120	120	120	120	120
*R* ^2^	0.553	0.449	0.515	0.473	0.537

***, **, and * means the statistical significance is at the 1, 5, and 10% accuracy level. The values in the brackets are the t statistics for each regression.

In Table 3, each study object has been estimated twice, the results in columns (x-1) (x = 1, 2, 3, 4, 5) are the estimation results without the controlled variables in model (6), and in the columns (x-2) are with them. From the columns (1–1) and (1–2), we are able to find that the coefficient of the DID term ($\hat{\beta }$) is statistically significant, and the significance level is improved to 5% level when the controlled variables have been reckoned in. If the controlled variables are unconsidered, the ERL policy can bring the reduction of −0.793% on the number of sick people in four pilot provinces on the whole; if considered, it can markedly reduce 1.029% of the people detected sickness generally. Compared with the industrial upgrading effect, the health promotion effect of the ERL is much higher both in significance and magnitude.

Besides the overall ERL effect, its effects on each pilot province are listed in the rest of the columns in Table 3. The results show that the ERL policy is thoroughly effective in Jiangsu Province and Hainan Province (as their DID coefficients $\hat{\beta }$ are significant in both two regressions), and is even larger in degree for them (their absolute values of $\hat{\beta }$ are larger than the overall's). However, in Chongqing Municipality, the ERL is effective to a limited degree because only column (5-1) appears significant. Hubei Province is thoroughly uninfluenced by the ERL as its insignificant results with or without the controlled variables [(3-1) and (3-2)]. Upon the results above, hypothesis H_2_ is basically true except for the Hubei province.

### Discussion on the Estimation Results

From the results of the health promotion effect in 4.2, the ERL policy shows a homogeneous favorable effect among the three out of four pilot provinces, which means the residents' health conditions can largely benefit from this ecological conservation policy. However, Hubei is the only unaffected province on health promotion, this is perhaps because the scale of its secondary industry was still ramping up rapidly rather than being controlled steadily, as its output value showed an average of 8.4% annual growth rate in 2016–2019, higher than that of 6.9, 5.3, and 6.9% in Jiangsu, Hainan and Chongqing (data source: National Bureau of Statistics of China). In fact, Hubei province has indeed introduced considerable chemical industries in the recent 10 years (45, 46), which may serve as a “pollution haven” for the eastern coastal areas which are seeking the relocation of the pollution-intensive industries.

Considering the lower development level in the Midwest, the urgency and the execution strength of the ecological protection there might give way to the economic growth and employment rate, where the manufacturing industry can bring them straightaway. In view of the bigger environmental capacity in terms of freshwater resources and land space, the Midwest has larger intake carrier space for the industry expansion. This implies that the ERL policy may play a role in industrial relocation from East to Midwest, and the results in 4.1 reinforce this view, where the industrial upgrading for Hubei and Chongqing are negatively affected by the ERL policy [see column (3-b) and (5-b) in Table 2], manifesting the ERL have enlarged their proportion of secondary industry.

Hainan province has benefited most from the ERL policy, as its health promotion degree and industrial upgrading degree are both the highest compared to the other three provinces. Two factors may explain this: one is that Hainan is an island province where the existing scale of the secondary industry is fairly less, its output value was equivalent to 2.6% of the Jiangsu and 6.5% of Hubei in 2015, bringing about much smaller inertia in industrial transformation; another is the stricter environmental rules in Hainan compared with mainland provinces, as Hainan has long been famous of the tourism, incurring more prohibitions on resource exploitation.

## Robustness Test and Dynamic Effect

### Parallel Trend Test

As mentioned in 3.1, a critical precondition of a practicable DID estimation is that the experimental group and the matched groups are sharing a similar parallel trend before 2015, the year of the policy implementation. Model (6) has shown effective results whose robustness is worth verifying. To verify the parallel trend before the year of ERL's implementation, I select 2014 (one year before 2015) as the base period, then generate the interaction terms of the time dummy variable and the experimental group. If the coefficients of these interactions are not significantly negative before the time of the exogenous impact, then the DID estimation passes the parallel trend test (33, 47, 48).

By referring to the existing studies (34, 37, 49, 50), an event study model to conduct the parallel trend test of ERL policy can be set as follows:


(7) $$\begin{array}{l} ln\left({Disease}_{it}\right)=\alpha +\overset{4}{\underset{k\geq -4,k\neq -1}{\sum }}\beta_{k}\cdot D_{it}^{k}+\overset{n}{\underset{j}{\sum }}\gamma_{j}\cdot {Control}_{it}\\ \;+\delta_{i}+\mu_{t}+\varepsilon_{it} \end{array}$$

Compared with single a DID term β·(*ELR*_*i*_ × *Post*_*t*_) in model (6), the second term in model (7) is changed into a sum term where a binary variable $D_{it}^{k}$ is set to signify the ERL policy, where *k* (−4 ≤ *k* ≤ 4) delegates the year distance to 2015 at year *t*. $D_{it}^{k}$ equals 1 only if the individual *i* is the ERL pilot province and *k* = *t*−2015, otherwise $D_{it}^{k}$ = 0 ($D_{it}^{-1}$ is not exist because 2014 is set as the base year). The other variables and coefficients are set the same as with that in the model (6).

**Figure 6:**
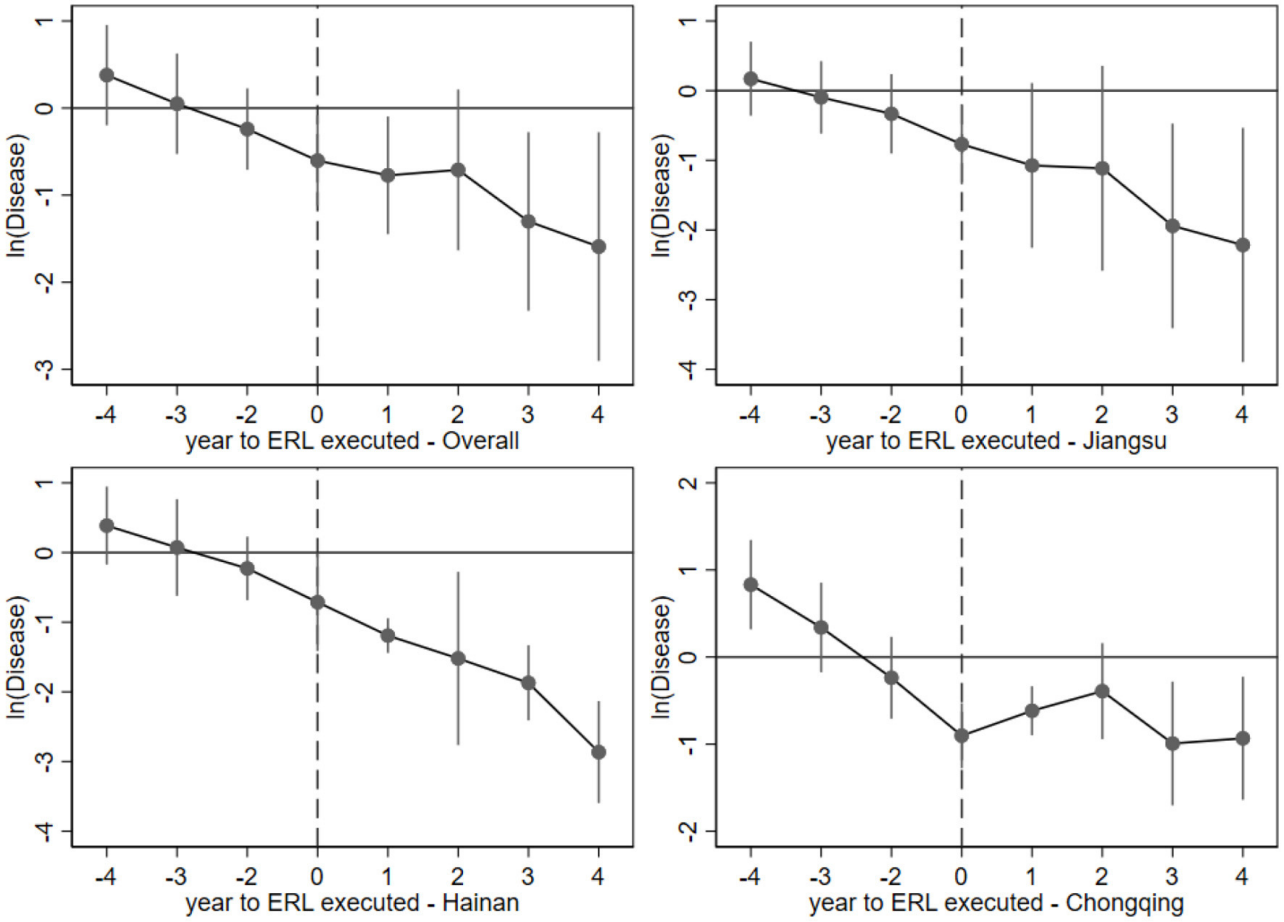
Parallel trend test results.

Using the same dataset in model (6), the result of the parallel trends test is plotted in Figure 6, where the year 2014 has been dropped because of the inexistence of $D_{it}^{-1}$, and Hubei province is untested because of its insignificant results in 4.2.

Knowing from Figure 6, the coefficients of the DID term in the event study are around zero before the ERL implementation year, and their 95% confidence interval (the vertical solid lines) covers value zero, which means they cannot reject the null hypothesis and can be zero. Hence, the pre-policy coefficients are basically statically insignificantly, while the coefficients after the ERL execution year are mainly negative and free from the null hypothesis. Based on the outcome of the event study, the DID model (6) conforms to the parallel trend, and it effectively proved that the ERL policy has reduced the number of diseases tested after 2015.

### Dynamic Effect Estimation

Although 4.1 has proved the fundamental effectiveness of the ERL policy in general and in three of the four pilot provinces, a time heterogeneity may exist among differently treated individuals. In other words, in the observation period 2015–2019, the significance and the magnitude of the health promotion effect may be varied among them. For a better understanding of the policy dynamic effect, the event study model (7) with its graphical results Figure 6 can provide a table of ERL's yearly impact below.

From the yearly DID estimation result displayed in Table 4, the overall and individual dynamic effects brought by the ERL policy are quantified. As the outcome of the overall test [column (1)], two coastal provinces, Jiangsu and Hainan [column (2) and (3)], present a similar dynamic effect of strengthening year by year. In contrast, the yearly coefficients for the inland Chongqing municipality show a slight inverted-V tendency after the ERL's execution year, indicating the health promotion effect may come into steady there. Moreover, the absolute values of the Chongqing's coefficients [column (4)] are all less than one, indicating that the magnitude of the ERL's health effect in Chongqing is also smaller.

**Table 4:** ERL's dynamic effect on health promotion.

**Explained variable**	**Overall (1)**	**Jiangsu (2)**	**Hainan (3)**	**Chongqing (4)**
*ERL_*i*_ × Post_2015_* ($\hat{\beta_{0}}=$)	−0.603** (−2.49)	−0.769** (−2.94)	−0.713** (−2.24)	−0.903*** (−5.32)
*ERL_*i*_ × Post_2016_* ($\hat{\beta_{1}}=$)	−0.773** (−2.45)	−1.073* (−2.00)	−1.192*** (−10.57)	−0.617*** (−4.82)
*ERL_*i*_ × Post_2017_* ($\hat{\beta_{2}}=$)	−0.710 (−1.65)	−1.114 (−1.67)	−1.520** (−2.69)	−0.392 (−1.56)
*ERL_*i*_ × Post_2018_* ($\hat{\beta_{3}}=$)	−1.302** (−2.72)	−1.941** (−2.91)	−1.871*** (−7.63)	−0.993** (−3.08)
*ERL_*i*_ × Post_2019_* ($\hat{\beta_{4}}=$)	−1.591** (−2.60)	−2.215** (−0.29)	−2.864*** (−8.61)	−0.933** (−2.91)
*Ln* * (H.Personnel)* (${\hat{\gamma }}_{1}=$)	4.496*** (3.19)	4.812** (2.76)	4.899** (2.76)	4.918** (2.75)
*ln(M.H.Asset)* (${\hat{\gamma }}_{2}=$)	−0.253 (−0.14)	−0.162 (−0.07)	−0.297 (−0.13)	−0.347 (−0.15)
Constant ($\hat{\alpha }=$)	−44.591** (−2.50)	−50.088** (−2.46)	−49.051** (−2.45)	−49.273** (−2.46)
Time fixed effects	Yes	Yes	Yes	Yes
Region fixed effects	Yes	Yes	Yes	Yes
Observations	150	120	120	120
*R* ^2^	0.498	0.535	0.544	0.546

***, **, and * means the statistical significance is at the 1%, 5%, and 10% accuracy level. The values in the brackets are the t statistics for each regression.

### Placebo Test

**Figure 7:**
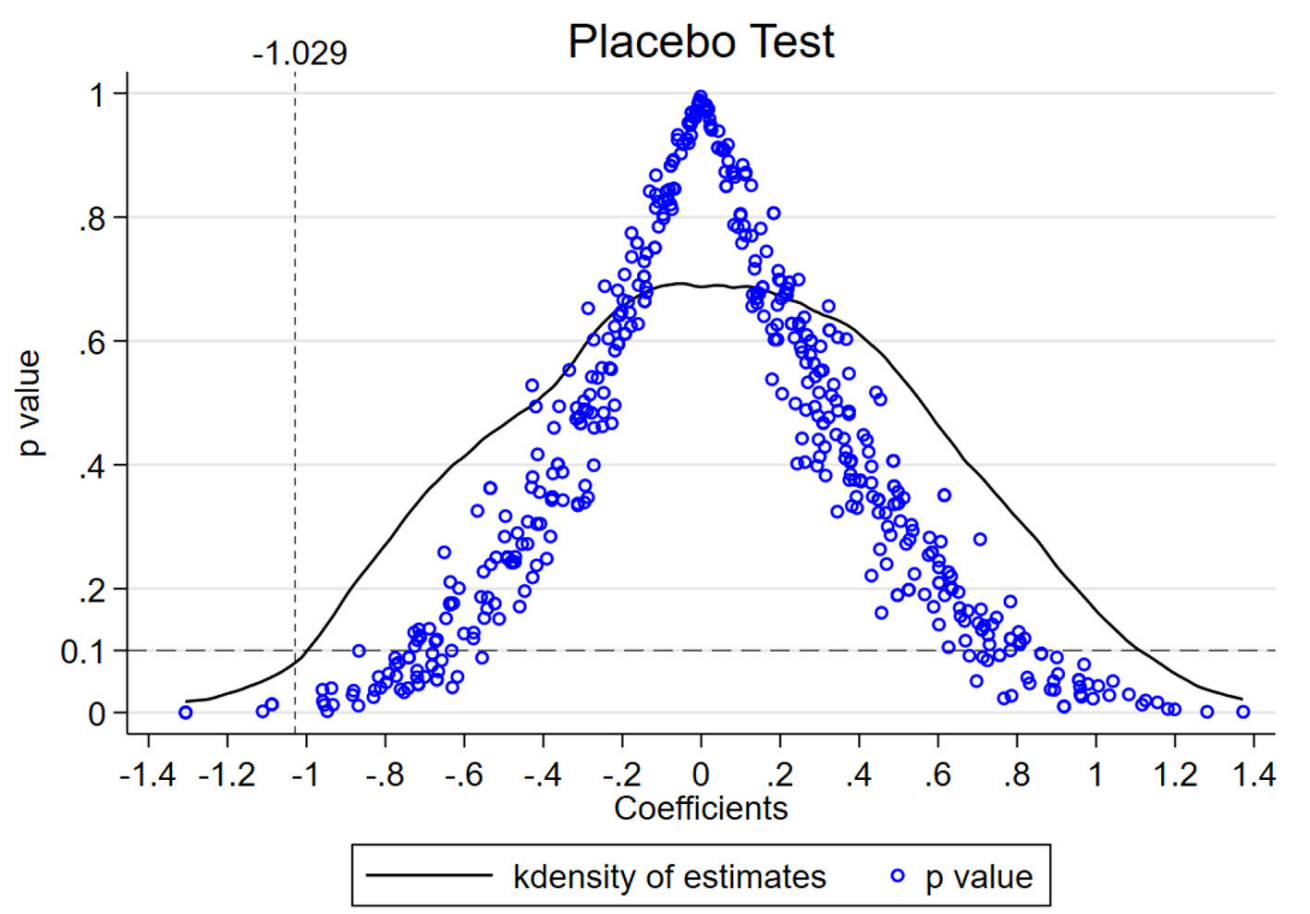
Placebo test result of DID model.

Another indispensable precondition of the DID model's effectiveness is the irreplaceability of the exogenous policy, which means the policy cannot affect the non-experimental individuals significantly and the explained variables are not affected by other unobserved variables (51, 52). This irreplaceability can be verified by a placebo test, the multiple assumptive tests where the individuals from the experimental group are substituted randomly by other non-experimental individuals (29). If the assumptive DID coefficients are distributed around zero (fail to reject the null-hypothesis) and most of their *p*-values are larger than 0.1 (fail to show the significance), then the placebo test is passed. To verify the irreplaceability of the ERL policy in model (6), a placebo test where the random process has been repeated 500 times is given, receiving the result in Figure 7.

Figure 7 plots the outcome of the placebo test, where the solid curve is the kernel density of the assumptive DID coefficients and the small circles are their *p*-values. Besides, −1.029 is the real DID coefficient estimated in Table 3, the *p*-value 0.1 denotes 10% of the significance level. Obviously, the coefficients in the placebo test are approximately in normal distribution around zero, and their *p*-values are basically larger than the 0.1, which means most of the assumptive estimations are insignificant and cannot reject the null hypnosis. Hence, the placebo test of the DID model (6) proves the robustness of the ERL's effects on health promotion.

## Conclusions

Although the policy of Ecological Red Lines (ERL) has reached some achievements, China would step up to utilize and expand the measures from it, including finalizing the regulations of environmental protections, perfecting the bottom lines for the ecological protections, and setting the ceilings of the resource exploitations. The industrial structure could also be upgraded by the innovation of green technologies or reasonable industrial policies, such as reinforcing the market negative list approach or setting the market-entry lists. The ERL policy is only one of China's policy mix in confronting environmental pollution, limiting harmful emissions, and achieving climate change targets. However, as a pilot policy, the ERL has indeed provided foundations for further climatic environment actions, such as Environmental protection tax law^3^, or Carbon Peak in 2030 and Carbon Neutrality in 2060^4^, which inherits and enlarges the concept of the eco-environmental protection.

This paper is devoted to evaluating a direct and an indirect effect brought by the Ecological Red Lines (ERL) policy implemented in four pilot provinces of China in 2015. By applying DID method, the direct effect indicated by the industrial upgrading is small (0.033% of the tertiary's output value) and even showed a negative effect on the inland Midwest provinces. The indirect effect which is indicated by the residents' health is generally at a much larger degree, as 1.029% of the residents' health conditions have been significantly improved. Moreover, the event study reveals that the ERL policy's effect on health promotion is increasing year by year as a whole, indicating its sustainability and cumulative achievements.

When decomposing the DID estimation into every single pilot province, the result of the industrial upgrading effect manifests a great heterogeneity among them, while the result of the health promotion effect turns out to be a favorable homogeneity. These differentiated outcomes indicate that most of the pilot provinces can reduce the adverse impact on people's health during the industrial restructuring, regardless of the change direction of tertiary industry's ratio. However, as the secondary industry has been expanded in the inland Midwest pilot provinces, the ERL policy may tend to be a cause of industrial relocation rather than reconstitution. Some negative signs have occurred in the Midwest regions: Hubei has not benefited from the health promotion effect significantly, Chongqing has benefited but has a fluctuant lasting effect compared to the two coastal pilot provinces which have shown incremental effects. The transference of the secondary industries from Eastern China to the Midwest may explain this, but more incisive provincial-level research on interregional industry relocations is needed in the future.

For the deficiencies of this study, the missed analysis of Shenyang City is first, which needs to be evaluated in the future city-level studies of the ERL in the future works. The possible incomplete selections of the explained variables may be another, as the environmental indicators are extensive and need more elaborate studies, which requires more comprehensive studies in further studies on the ERL. Moreover, the combined effects of the ERL policy and other environmental policies such as “Action Plan of Air Pollution Prevention and control” have not been embodied in this paper, which can be verified with derivative measurement models in future works, such as triple difference (DDD) models. In addition to this, the ERL may have a spillover effect on the adjacent regions out of the pilot places, future research may detect this effect with spatial econometrics.

Based on the above conclusions, some constructive suggestions can be given as: (1) For the economically developed and densely populated eastern coastal regions, the cover range of the ERL policy can be extended; (2) For the inland Midwest regions with the lagging stage of economic development, some eco-compensation measures should be taken if the ecological protection policies are strict; (3) To maintain an eco-friendly sustainable development without ignoring the development demands, some flexible methods are recommended, such as improving green productivity for manufacturing, building ecological protection facilities for waste treatment, and planning reasonable residence zones for the people's health; (4) In view of the heterogeneous effects between coastal and inland regions of the ERL' trial implementation, some distinct protective or restrictive measures are essential, which may be better for the development needs of the less advanced regions. (5) Balancing ecological protection and economic growth is still a dilemma for developing regions, and a “one-size-fits-all” solution is inexistent, therefore, moderate and continuable strategical steps are of the essence, such as the long-term investments of green technologies, reasonable fiscal subsidies on industrial transformations, and the capitalization of the natural resources. Finally, the modifiability and the transparency of the long-run development of the ERL policy is the basic guarantee of sustainable development in all aspects of society.

## Data Availability Statement

The original contributions presented in the study are included in the article/Supplementary Material, further inquiries can be directed to the corresponding author/s.

## Author Contributions

PY: conceptualization, project administration, data acquisition, methodology, formal analysis, writing—original draft, and writing—review and editing.

## Funding

This research is funded by Hainan University Research Start-up Fund.

## Conflict of Interest

The author declares that the research was conducted in the absence of any commercial or financial relationships that could be construed as a potential conflict of interest.

## Publisher's Note

All claims expressed in this article are solely those of the authors and do not necessarily represent those of their affiliated organizations, or those of the publisher, the editors and the reviewers. Any product that may be evaluated in this article, or claim that may be made by its manufacturer, is not guaranteed or endorsed by the publisher.

## Footnotes

^1^Shenyang is not included in our analysis because of its city-level size and feature, hence it is improper to share the same comparable provincial-level groups with other four provinces, future work may carry through a city-level evaluation on Shenyang.

^2^Considering the table space and secondary status, the estimation results without the controlled variables for model (4) and (5) are moved into the Supplementary Material.

^3^*Environmental Protection Tax Law of P.R. China*. Available online at: http://www.gov.cn/zhengce/content/2017-12/30/content_5251797.htm (accessed December 30, 2017).

^4^*The Guidelines on Full Implementation Works on the Idea of Development for Peaking Carbon and Carbon Neutrality*. Available online at: http://www.gov.cn/zhengce/content/2017-12/30/content_5251797.htm (accessed October 24, 2021).
