# Supplementary material for: Policy Effects of Ecological Red Lines on Industrial Upgrading and Health Promotion: Evidence From China Based on DID Model
Source: Front Public Health. 2022 Mar 8;10:844593. doi: 10.3389/fpubh.2022.844593 (PMC8957215; doi:10.3389/fpubh.2022.844593)
Supplement: Supplementary file 1 [file Table_1.docx]

Policy effects of Ecological Red Lines on industrial upgrading and health promotion: Evidence from China based on DID model

Supplemented Tables

Penghao Ye^1*^

^1^ School of Economics, Hainan Open Economy Research Institute, Hainan University, Haikou 570228, Hainan Province, China

*** Correspondence:**Penghao Ye
paulyph@hainanu.edu.cn

------------------Below are the tables in the Supplementary Material----------------------

| **Supplemented Table 1a** Policy estimation results of industrial upgrading (without control variables) | | | | | |
| --- | --- | --- | --- | --- | --- |
| **Explained variable** | **Overall**  *ln(Tertiary)*  (1-a) | **Overall**  *Update*  (1-b) | **Jiangsu**  *ln(Tertiary)*  (2-a) | **Jiangsu**  *Update*  (2-b) | **Hubei**  *ln(Tertiary)*  (3-a) |
| *DID* = *ERL*_i_×*post_t_*  ($\hat{\beta}$=) | 0.061***  (3.30) | 0.034  (0.84) | 0.018  (0.50) | -0.045  (-0.71) | 0.113**  (3.16) |
| *ln(Estate)*  ($\hat{\gamma}_{1}$=) | - | - | - | - | - |
| *ln(Energy)*  ($\hat{\gamma}_{2}$=) | - | - | - | - | - |
| Constant  ($\hat{\alpha}$=) | 8.139***  (628.39) | 0.961***  (34.36) | 8.184***  (533.23) | 0.982***  (32.29) | 8.102***  (516.61) |
| Time Fixed Effects | Yes | Yes | Yes | Yes | Yes |
| Region Fixed Effects | Yes | Yes | Yes | Yes | Yes |
| Observations | 150 | 150 | 120 | 120 | 120 |
| R^2^ | 0.983 | 0.752 | 0.980 | 0.780 | 0.980 |
| Note: ***, **, and * means the statistical significance is at the 1%, 5%, and 10% accuracy level. The values in the brackets are the *t* statistics for each regression. | | | | | |

| **Supplemented Table 1b** Policy estimation results of industrial upgrading (without control variables) - continued table of Supplemented Table 2a | | | | | |
| --- | --- | --- | --- | --- | --- |
| **Explained variable** | **Hubei**  *Update*  (3-b) | **Hainan**  *ln(Tertiary)*  (4-a) | **Hainan**  *Update*  (4-b) | **Chongqing**  *ln(Tertiary)*  (5-a) | **Chongqing**  *Update*  (5-b) |
| *DID* = *ERL*_i_×*post_t_*  ($\hat{\beta}$=) | -0.033  (-0.53) | 0.047  (1.33) | 0.332***  (4.71) | 0.066*  (1.89) | -0.119**  (-1.88) |
| *ln(Estate)*  ($\hat{\gamma}_{1}$=) | - | - | - | - | - |
| *ln(Energy)*  ($\hat{\gamma}_{2}$=) | - | - | - | - | - |
| Constant  ($\hat{\alpha}$=) | 0.896***  (32.64) | 7.944***  (510.559) | 0.980***  (31.77) | 8.058***  (523.02) | 0.914***  (33.02) |
| Time Fixed Effects | Yes | Yes | Yes | Yes | Yes |
| Region Fixed Effects | Yes | Yes | Yes | Yes | Yes |
| Observations | 120 | 120 | 120 | 120 | 120 |
| R^2^ | 0.782 | 0.980 | 0.789 | 0.980 | 0.773 |
| Note: ***, **, and * means the statistical significance is at the 1%, 5%, and 10% accuracy level. The values in the brackets are the *t* statistics for each regression. | | | | | |
